# Current State and Future Perspectives of Artificial Intelligence for Automated Coronary Angiography Imaging Analysis in Patients with Ischemic Heart Disease

**DOI:** 10.1007/s11886-022-01655-y

**Published:** 2022-03-28

**Authors:** Mitchel A. Molenaar, Jasper L. Selder, Johny Nicolas, Bimmer E. Claessen, Roxana Mehran, Javier Oliván Bescós, Mark J. Schuuring, Berto J. Bouma, Niels J. Verouden, Steven A. J. Chamuleau

**Affiliations:** 1 Amsterdam University Medical Centers—Location VU Medical Center, Department of Cardiology, University of Amsterdam, Amsterdam, The Netherlands; 2 Amsterdam University Medical Centers—Location Academic Medical Center, Department of Cardiology, University of Amsterdam, Amsterdam, The Netherlands; 3 The Zena and Michael A. Wiener Cardiovascular Institute, Icahn School of Medicine at Mount Sinai, One Gustave L. Levy Place, Box 1030, New York, NY 10029-6574 USA; 4 Department of Interventional X-Ray, Philips Healthcare, Best, The Netherlands; 5 Amsterdam Cardiovascular Sciences, Amsterdam University Medical Centers—Location Academic Medical Center, University of Amsterdam, Amsterdam, The Netherlands

**Keywords:** Coronary angiography, Coronary stenosis, Artificial intelligence, Deep learning, Image processing

## Abstract

**Purpose of Review:**

Artificial intelligence (AI) applications in (interventional) cardiology continue to emerge. This review summarizes the current state and future perspectives of AI for automated imaging analysis in invasive coronary angiography (ICA).

**Recent Findings:**

Recently, 12 studies on AI for automated imaging analysis In ICA have been published. In these studies, machine learning (ML) models have been developed for frame selection, segmentation, lesion assessment, and functional assessment of coronary flow. These ML models have been developed on monocenter datasets (in range 31–14,509 patients) and showed moderate to good performance. However, only three ML models were externally validated.

**Summary:**

Given the current pace of AI developments for the analysis of ICA, less-invasive, objective, and automated diagnosis of CAD can be expected in the near future. Further research on this technology in the catheterization laboratory may assist and improve treatment allocation, risk stratification, and cath lab logistics by integrating ICA analysis with other clinical characteristics.

## Introduction

Artificial intelligence (AI) has an emerging role in healthcare in general, and the same holds for cardiology specifically, with numerous solutions in cardiac imaging modalities on image acquisition and reconstruction, diagnosis, and prognosis [1]. For example, AI applications are now being utilized to accelerate acquisition and reduce reconstruction time of cardiac MRI, to automate disease classification in echocardiography, and to improve conventional risk prediction models based on coronary CT angiography features [2–5]. Despite growing applications in general cardiology, the role of AI in automated analysis of invasive coronary angiography (ICA) is less clear. ICA is an indispensable step in the diagnosis of coronary artery disease (CAD) in symptomatic patients [6]. This invasive imaging modality assesses the severity of stenoses by X-ray imaging of contrast-filled coronary arteries. In case of significant CAD, a multidisciplinary heart team decides on an appropriate treatment strategy, either conservative management or percutaneous or surgical revascularization. The heart team assessment is largely based on ICA assessment in combination with clinical parameters. Furthermore, percutaneous coronary interventions (PCIs) are guided by ICA for identification of target lesions; determining wiring, lesion preparation, and stenting strategies; and evaluation of procedural success based on residual stenosis, absence of significant dissection, and flow [6].

After a general introduction of AI (as an application), we summarize the current state of AI for ICA imaging analysis and discuss its clinical implications for diagnosis, (real-time) treatment guidance, and risk stratification. We conclude this review with a discussion of its current limitations and future perspectives.

## Artificial Intelligence: a Deeper Understanding

Artificial intelligence (AI) has become a collective term for applications that perform complex tasks that previously required human intelligence. Machine learning (ML), a subfield of AI, is performing complex tasks by learning from experience. Training of an ML algorithm creates an ML model, which represents what was learned by the ML algorithm to make predictions on new data. Most common ML applications in cardiac imaging can be broadly subdivided into two categories: supervised learning and unsupervised learning. In supervised learning, categorized data are used to classify unseen data. An example of supervised learning is the training of ML algorithms to predict a patient’s response to certain treatment. In unsupervised learning, ML algorithms are trained to find patterns or conclusions through unlabeled training data. A well-known unsupervised learning method is clustering in which data/patients are grouped on similarity, for example, to identify distinct clinical subgroups of patients which may benefit from targeted therapy [7, 8].

**Fig. 1:**
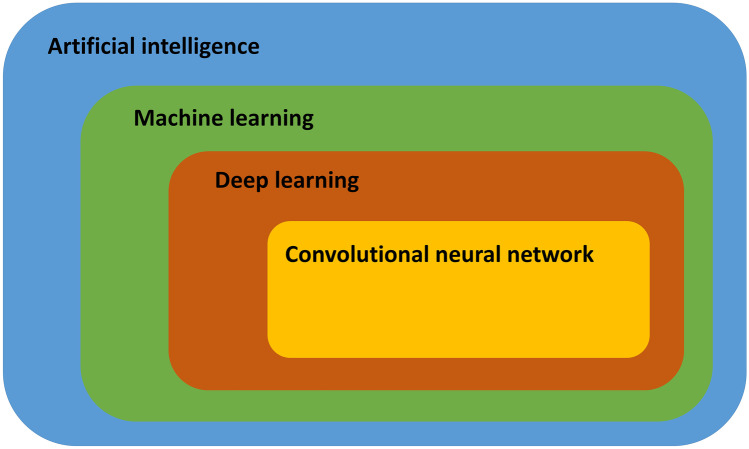
Conceptual framework of artificial intelligence with its subfields machine learning and deep learning

Deep learning (DL) is a subfield of ML in which multilayered neural networks are trained to learn a supervised or unsupervised task. A neuron is a mathematical function that provides an output based on the input. During training, weights of the neurons in a neural network are optimized to map the input(s) to a desired output. Feature selection is an important processing step to select relevant input variables before training an AI algorithm. The selection of features that are most related to the outcomes reduces the complexity of the model and increases training speed. Moreover, noisy and redundant features are eliminated which increases the performance of the model. In contrary to ML, neural networks can automatically select features. Therefore, DL can be trained directly on unstructured data like text, sound, video, and images. DL is a computationally expensive subfield of ML and requires large datasets to avoid generalization errors [9]. The number of neurons, number of layers, and connections between neurons determine the complexity and architecture of a DL algorithm [10]. The convolutional neural network (CNN) is a class of DL (Fig. 1) that is widely used for imaging applications. Trained CNNs have the ability to detect and classify distinctive features (e.g., edges of anatomical structures) on images, for example, to classify views of echocardiograms [3, 11].

ML models are reported with a variety of metrics, which are selected for the ML application. Examples of metrics are the F1 score, accuracy, sensitivity, dice similarity coefficient (DSC), area under the receiver operating characteristic curve (AUC), and concordance statistic (C). These metrics are explained in detail elsewhere [12].

## Automated Interpretation of ICA

### Search and Selection Strategy

A literature search was performed in the following databases: PubMed, Web of Science, Embase, and Google Scholar. The databases were searched in the publication period July 30, 2011 until July 30, 2021 with the following combined terms: (1) Coronary angiography AND (2) Artificial intelligence NOT (3) computed tomography. The exact search strategy is shown in the Appendix. Relevant studies were selected using machine learning–driven selection software called ASReview, which is further explained in the Appendix [13]. Relevant peer-reviewed articles were included if artificial intelligence models were developed on coronary angiography imaging data. Articles that solely focused on automated segmentation without other AI applications, reviews, and letters to editor were excluded. Records classified as non-relevant and reference lists were examined to find additional relevant studies. The search strategy resulted in 1335 studies. After deduplications and screening on title and abstract, 12 studies were included. A flow chart of study inclusion is shown in Fig. 3 (see Appendix). The included studies reported on ML models for the following (diagnostic) applications: automated frame selection, segmentation, lesion assessment, and functional assessment of coronary flow. These applications will be summarized after a short introduction into ICA interpretation in daily clinical practice and its current limitations.

**Fig. 2:**
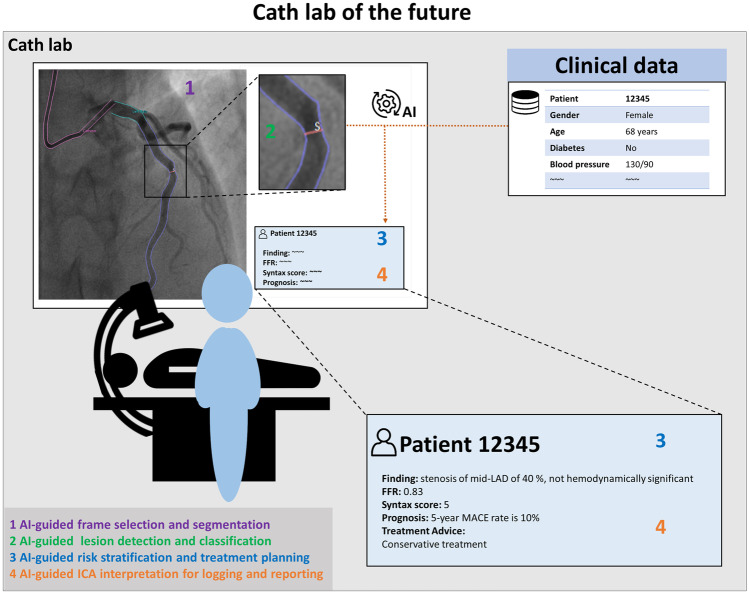
Example of future, automated invasive coronary angiography analysis: artificial intelligence (AI) for automated quantitative coronary angiography (QCA) with FFR estimation, (syntax-based) clinical risk scoring and reporting

### ICA Interpretation in Daily Clinical Practice

The interpretation of ICA is highly standardized and consists of the assessment of multiple components including coronary flow (Thrombolysis in Myocardial Infarction (TIMI) flow), lesion severity (percentage of stenosis and length), and other characteristics such as the presence of thrombi and calcifications. Despite standardized interpretation, ICA has well-known limitations. Coronary arteries are three-dimensional (3D) structures that are captured in two-dimensional (2D) images, which may result in overlap, foreshortening, and difficulty in assessing true (3D) stenosis grade. ICA image quality is further affected by low-dose radiation, commonly used in these procedures, heart motions and X-ray absorbing tissues (e.g., ribs and vertebrae), which leads to low signal-to-noise ratio, low-contrast regions, and blur [14–18]. These limitations make ICA prone to subjective interpretation, which may have important diagnostic and therapeutic repercussions [19, 20].

### Frame Selection

ICA analysis is preferably performed during the end-diastolic phase of the cardiac cycle to minimize coronary artery motion and herewith prevent artifacts. Selection of contrast-filled frames in end-diastolic phase is a manual and time-consuming task, which lends itself for automation. Researchers demonstrated that a CNN could be trained on 56,655 coronary angiograms from 6820 patients to detect the end-diastolic phase [21•]. Electrocardiography signals were used as ground truth. The CNN yielded good performance with an F1 score of 0.995. Instead of selecting one frame, other investigators trained a CNN on 90 ICA sequences in which three consecutive, contrast-filled frames were selected [22]. The rationale of training with three consecutive frames was to reduce the number of false-positive observations of significant stenosis. These nonexistent stenoses are often visible on a single frame and caused by heterogeneity of contrast filling, curved vessels, coronary motion, or background noise. With an accuracy of 0.87 to select contrast-filled frames, the network performed better than conventional segmentation-based methods.

### Segmentation

Selected frames can be segmented, which is a process to classify pixels as coronary arteries or irrelevant structures. Training an algorithm to identify relevant structures is crucial for detecting, localizing and classifying coronary lesions. To date, most studies on automated ICA image analysis have trained DL algorithms to automatically segment coronary arteries in coronary angiography [15, 16, 23–28]. Segmented coronary arteries can be partitioned into smaller structures based on, for example, location or anatomy. Recently, Du et al. trained a neural network (cGAN [29]) on 12,323 angiograms collected from 2834 patients to label coronary arteries into 20 segments [30••]. Although not specified, this 20-segment model looks similar to the segment model of the globally accepted SYNTAX (Synergy Between Percutaneous Coronary Intervention With Taxus and Cardiac Surgery) score [30••]. The SYNTAX score is an objective tool to grade complexity of CAD and guides decision-making between PCI and coronary artery bypass graft (CABG). The recognition model was tested using an additional 1050 angiograms and showed a recognition accuracy of 98% and sensitivity of 85%. Both training and test data were collected from a single medical center. Aforementioned studies on automated frame selection and segmentation are shown in Table 1.

**Table 1:** Studies on artificial intelligence for automated coronary angiography imaging analysis (if multiple AI architectures were valuated, the best performing model was reported)

**First author**	**Publication year**	**AI application**	**Data**	**Classifier**	**Metric value**	**Labeled/annotated by**
**Segmentation**						
Du T [30••]	2021	Segmentation	20,612 ICA images of 10,073 patients	cGAN	ACC = 98%, SE = 85%	QA
Zhao C [42]	2021	Segmentation	314 ICA images of 99 patients	CNN	DSC = 0.89	EC
**Frame selection**						
Ciusdel C [21•]	2020	End-diastolic frame detection	56,655 ICA sequences of 6820 patients	CNN	F1 = 99.5%	ECG
Wu W [22]	2020	Segmentation for frame selection	148 ICA sequences of 63 patients	CNN	SMT: visually, FS: ACC = 0.87	EC
**Lesion detection, localization, and classification**						
Moon JH [48]	2021	Lesion detection, localization, and classification	452 ICA images	CNN	AUC = 0.96	QA and EC
Danilov VV [50••]	2021	Lesion detection and localization	8325 ICA images of 100 patients	CNN	F1 = 0.96	EC
Du T [30••]	2021	Lesion detection, localization, and classification	20,612 ICA images of 10,073 patients	CNN	F1 = 0.80–0.85	QA
Zhao C [42]	2021	Lesion detection, localization, and classification	314 ICA images of 99 patients	CNN	TPR = 0.68, PPV = 0.70	EC
Pang K [47]	2021	Lesion detection and localization	166 ICA sequences	CNN	F1 = 0.88	QA
Chen S [44]	2020	Lesion detection and classification	21,631 ICA sequences of 14,509 patients	CNN	F1 = 0.91 − 0.97	NS
Wu W [22]	2020	Lesion detection	148 ICA sequences of 63 patients	CNN	F1 = 0.83	EC
Yabushita H [46]	2020	Lesion detection	1838 ICA sequences 199 patients	CNN	C = 0.61	EC
Ovalle-Magallanes E [49]	2020	Lesion detection	250 ICA images	CNN	F1 = 0.95	NS
Liu X [43]	2019	Lesion detection, localization, and classification	2059 ICA images	CNN	F1 = 0.89, AUC = 0.98	EC
**Functional assessment of coronary flow**						
Roguin A [60•]	2021	Fractional flow reserve estimation	31 patients	NS	SE = 88%, SP = 93%	EC
Cho H [59]	2019	Fractional flow reserve estimation	1717 patients	XGBoost	AUC = 0.87	NS

*ACC* accuracy, *AUC* area under curve, *cGAN* conditional generative adversarial network, *CNN* convolutional neural network, *DSC* dice similarity coefficient, *EC* experienced cardiologist, *F1* F1 score, *ICA* invasive coronary angiography, *NS* not specified, *SE* sensitivity, *SP* specificity, *SMT* segmentation, *TPR* true-positive rate, *PPV* positive predictive value, *QA* qualified analyst

Despite limitations of ICA images on image quality, aforementioned studies show that it is feasible to train an AI algorithm to select frames of interest and automatically segment coronary arteries in a proper fashion.

### Lesion Detection, Localization, and Classification

Several efforts have been made to improve ICA interpretation. Quantitative coronary angiography (QCA) software is already available for over three decades and can provide objective and quantitative assessment of anatomical lesion severity. However, QCA requires manual, time-consuming input and has therefore not been widely implemented into clinical practice [31, 32]. In recent years, software has been developed to reduce noise and improve detection of stenosis in coronary arteries [14, 33–36]. However, these methods are often computationally expensive [22, 37–39], semi-automatic, and have long processing times [22, 39–41].

The inter- and intra-observer variability of visual assessment of lesions by clinicians could be minimalized if lesion detection, localization, and classification are automated. Du and colleagues trained a CNN on 6239 lesions to improve lesion detection and categorize lesions into stenotic lesions, total occlusions, calcific lesions, and the presence of thrombus or dissection. Internal validation of CNN performance on 1000 ICA images demonstrated F1 scores between 0.80 and 0.85. Other studies performed classification on the degree of stenosis (mild, moderate, severe), or elements of SYNTAX, such as the presence and type (blunt/tapered stump) of total occlusion with moderate to good results (Table 1) [42–44].

Large amounts of labeled data are needed to train an algorithm that generalizes well to unseen data [45]. In a study by Yabushita et al. training on 199 ICA images resulted in modest performance (C = 0.61) to detect the presence of clinically significant coronary stenoses [46]. In the setting of lower volume datasets, diagnostic accuracy of ML models could be enhanced by several strategies. As an example, training of CNNs on sequences of frames improved the rate of false-positive stenoses. Researchers demonstrated that by incorporating temporal information, F1 scores increased by 30–40% [22, 47]. Transfer learning and data augmentation are other strategies that can increase performance. In transfer learning, an AI model, already being trained for another task, will be further made ready for other purposes. Data augmentation is a technique to increase the amount of data without collecting new data. For example, an AI model pre-trained on a large image database (ImageNet) was further trained on 45,125 frames to localize stenoses with a cut-off of 50% in the right coronary artery [48].The ICA frames were derived from 452 ICA frames by cropping and pixel intensity value adjustments. Validation on an external dataset yielded an excellent AUC of 0.96. Other researchers also demonstrated the power of pre-training by employing an automated lesion detection CNN by means of training on only 125 images. Despite the limited amount of training data, F1 score was as high as 0.95 [49].

Real-time detection of coronary stenoses can facilitate operators to identify lesions that might have otherwise been unnoticed. However, the processing time of such an AI model is an important constraint which is often not reported in studies. As a fact, a better ML model accuracy often means a higher complexity of its architecture and processing time [50••]. Real-time application of AI should not result in time delays, which may affect the outcome of patients [51]. Therefore, there should be a trade-off between accuracy and speed in deployed models [50••].

In summary, there has been a great deal of progression in automated detection and classification of CAD in the last decade. These developments are attributable to gains in computing power, advances in ML algorithms, and availability of large ICA datasets [52]. Automated detection and classification of CAD may provide physicians objective and reproducible information and may prevent significant lesions to be missed [22, 30••, 50••]. All studies on automated lesion detection, localization, and classification are shown in Table 1.

### Functional Assessment of Coronary Flow

Functional sufficiency of coronary flow and plaque characterization are fundamental elements that guide treatment decisions but cumbersome features to assess on ICA [20, 53]. There is a discrepancy between visual assessment and intracoronary pressure measurement for assessment of functional sufficiency of coronary flow [54, 55]. Therefore, intracoronary pressure measurements are performed to assess whether a stenosis is functionally significant and herewith causes myocardial ischemia [56]. Fractional flow reserve (FFR) is the most used metric and records the mean distal coronary pressure divided by the mean proximal pressure during maximal hyperemia. Although evidence shows that FFR-based decision-making for revascularization leads to improved cardiovascular outcomes [57, 58], the FFR technique has its limitations. Major limitations of this technique are its invasive nature and necessity of use of costly pressure wires. In addition, prolonged procedural time and operator’s preference for visual assessment limit the implementation of routine intracoronary measurements during ICA [54, 55, 59, 60•]. To overcome these limitations, quantitative flow ratio (QFR) applications have been developed to add functional assessment to anatomic imaging analysis. QFR is a non-invasive method to calculate functional sufficiency based on 3D-angiographic reconstruction and computational fluid dynamics [61, 62]. To date, QFR analysis is not readily available for daily clinical practice at the catheterization laboratory (cath lab) and requires computationally expensive post-processing.

AI-based FFR estimation is likely to have less processing time compared to QFR estimation based on computational fluid dynamics, as demonstrated by studies on AI-based FFR estimation on coronary CT [63, 64]. Recently, a ML model (XGBoost [65]) was developed on data of 1501 patients to classify intermediate lesions as having a FFR ≤ 0.8 or FFR > 0.8 [59]. Feature selection resulted in a set of 12 features including body surface area, sex, and 10 features extracted from ICA images (lengths and diameters of lumen and stenosis). Evaluation of this classification model on an external dataset yielded an AUC of 0.87. More recently, a feasibility study was conducted to compare novel AI-based FFR software to invasive FFR measurements [60•]. This software, called AutocathFFR, was able to detect coronary lesions and predict their FFR value without coronary artery annotation or view selection. The diagnostic value of AutocathFFR to classify a lesion as functional significant was evaluated in 31 patients, with the left anterior descending artery as the most frequent target (25 of 31 patients). The sensitivity, specificity, positive predictive value, and negative predictive value were 0.88, 0.93, 0.94, and 0.87, respectively. These results are similar to the performance of QFR and demonstrate the feasibility for automated FFR estimation. All studies on AI-based functional assessment of coronary flow are shown in Table 1.

Although automated assessment of FFR directly from ICA images has potential to speed up procedures, studies investigating real-time QFR-based PCI versus standard of care (i.e., FFR-guided PCI) are still ongoing. Successful introduction of QFR-based coronary treatment might eventually reduce over- and under-treatment. Furthermore, the need to perform intracoronary hemodynamic measurements will diminish, which might result in lower incidence of complications and lower healthcare costs.

## Limitations and Challenges in Development of Automated ICA Analysis

AI has the potential to increase diagnostic performance and support clinicians in therapeutic decision-making by automatically assessing the extent and functional significance of CAD in the cath lab. However, multiple barriers have to be overcome before these models can be implemented into clinical practice.

### AI Bias

A key challenge in development of smart technology is to work toward generalizable AI applications, which are externally validated and trained on large and variable patient populations from multiple centers [66]. However, because most studies are proof-of-concept studies, external validation is often not performed [67]. Results of only three out of the 12 studies mentioned in Table 1 have been externally validated [46, 48, 59]. Therefore, datasets should be shared between research centers or made open-access according to FAIR (Findability, Accessibility, Interoperability, and Reusability) data principles [68]. To avoid the risk of algorithmic bias, subgroup analysis on populations (e.g., age, ethnicity, sex, and medical center) should be performed. These analyses will show whether population subgroups were underrepresented in the training data and whether more data for training should be collected [66].

### AI Interpretability

Diagnosis and therapeutic decision making has a tremendous impact on clinical outcomes in the cath lab. There is a need for AI applications in which algorithmic decisions are clearly explained (explainable AI), so that eventual inaccurate analysis can be back traced. However, algorithmic decisions are often difficult to understand due to its “black box” nature. Currently, ongoing research on explainable AI is likely to enhance trust among users and facilitate adoption of AI applications [69-71].

### AI in ICA versus Other Cardiac Modalities

To our best knowledge, no studies have been published on AI applications developed for treatment guidance, risk stratification, or prognosis based on ICA imaging. This is in contrast to the cardiac imaging modalities echocardiography, coronary computed tomography angiography (CCTA) and magnetic resonance imaging (MRI), in which the first AI applications to predict prognosis have emerged [5, 72–76]. This development delay could be explained by several factors. In ICA, registration of 3D structures in 2D images results in overlapping coronary arteries, which hinder AI models to find coronary artery specific features. This overlap in coronary arteries with anatomical variation and heterogeneity among operators regarding X-ray beam projections results in the need for large ICA datasets in order to develop a well-performing AI model in the setting of ICA. Pre-processing (selection, segmentation, classification) of these datasets is a time-consuming and tedious process. Another possible explanation is that ICA has a different role in the diagnostic and treatment trajectory of patients with (suspected) ischemic heart disease compared to other, noninvasive imaging modalities. Its invasive nature makes ICA a second-line diagnostic test, only applied in patients with high a priori probability of CAD. This might favor development of AI models for other cardiac imaging modalities earlier in the diagnostic trajectory compared to ICA.

## Future Perspective of AI in the Cath Lab

### Growing Healthcare Utilization

ICA has numerous important challenges to overcome in the next decades. Growing burden of cardiovascular disease is likely to increase the number of interventions being performed. As a consequence, workload and healthcare costs will further increase. Without smart solutions, personnel exhaustion and delayed or cancelled interventions will jeopardize quality of care [77]. AI as a smart technology has the potential to alleviate pressure on healthcare services in general and to improve cath lab diagnoses, treatment, and logistics in particular.

### AI-Guided ICA Interpretation for Logging and Reporting

The amount of administrative work of employees of the cath lab is increasing swiftly and ensures that time available for efficient patient care is minimized [78]. Automated logging and reporting of procedures by automated, AI-based ICA interpretation can reduce this administrative burden (see Fig. 2). Some examples of mundane tasks that may be reported automatically are the location and significance of the lesion and whether implants (e.g., stents) have been placed.

### AI-Guided Treatment Planning

AI models that allow accurate and fast evaluation of coronary anatomy and noninvasive functional sufficiency will offer an opportunity to further develop AI technology that will be able to guide real-time PCI procedures. Peri-procedural analysis of ICA images, including automated functional assessment, could optimize PCI outcomes by providing a lesion-specific recommendation on a revascularization strategy, eventually with advice on stent size, length, location, and preferred strategy (Fig. 2). After stenting, automated measurements on the proportion of stent under expansion and hemodynamic function may inform the operator and patient about the expected short- and long-term outcome [79, 80].

### AI-Guided Risk Stratification and Prognosis

The SYNTAX score, and subsequently the SYNTAX II score (which adds clinical characteristics to the anatomical assessment of the coronary tree), are examples of available stratification tools to guide clinical decision-making in complex CAD. However, these scores are time-consuming (5–10 min) to calculate and therefore underutilized in daily clinical practice, especially during ICA. Improved SYNTAX-like scores, integrating automated AI-based ICA imaging analysis and key clinical characteristics (extracted from electronic patient dossiers by intelligent and complex AI applications), might improve risk stratification of the individual patient and herewith enhance patient-tailored treatment, and ultimately prognosis (Fig. 2).

### Other AI-Guided Applications

Other AI applications in ICA, beyond the scope of this review, may reduce radiation exposure by focusing on image acquisition and reconstruction [81]. In addition, AI in intracoronary imaging (e.g., intravascular ultrasound (IVUS), optical coherence tomography (OCT), near-infrared spectroscopy (NIRS)) may lead to improved identification of truly vulnerable coronary plaques and may further elucidate the genesis of in-stent restenosis [82]. Ultimately, AI-based integration of upstream diagnostic modalities (i.e., CCTA), ICA, and intracoronary imaging may lead to optimal outcomes after PCI.

## Conclusion

The cath lab is on the verge of a new era in which AI-based state-of-the-art models are being developed for diagnostic and treatment guidance, optimized risk stratification, and automated cath lab logistics. We are still in an early stage of development, as most models are constructed on single-center datasets and external validation is often lacking. Large multicenter datasets are necessary to develop more generalizable models and cath lab field-labs, mirrored to real-life cath labs, are indispensable to readily test them.

## Figures and Tables

**Fig. 3:**
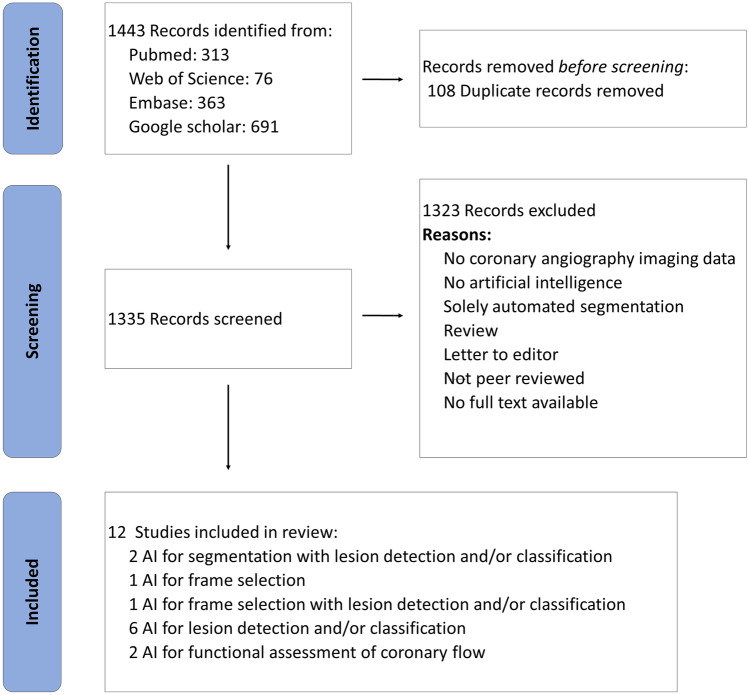
Flowchart of study inclusion

## Appendix

### Search Strategy

#### PubMed

("Coronary Angiography"[Mesh] OR "coronary angiogra*"[tiab] OR "Coronary Vessels"[Mesh] OR "Coronary Stenosis"[Mesh] OR "coronary stenosis"[tiab]) AND ("Artificial Intelligence"[Mesh] OR "Artificial Intelligence"[tiab] OR "AI"[tiab] OR "machine learning"[tiab] OR "deep learning"[tiab] OR "neural network"[tiab] OR "CNN"[tiab] OR "automatic image analysis"[tiab] OR "computer-aided diagnosis"[tiab]) NOT ("CT"[ti] OR "tomogr*"[ti])

#### Web of Science Search

TS = ("Coronary Angiography" OR "coronary angiogra*" OR "Coronary Vessels" OR "coronary stenosis") AND TI = ("Artificial Intelligence" OR "AI" OR "machine learning" OR "deep learning" OR "neural network" OR "CNN" OR "automatic image analysis" OR "computer-aided diagnosis") NOT TI = ("CT" OR "tomogr*")

#### Embase

(((coronary angiography OR coronary vessels OR coronary sten*).ti,ab OR exp coronary angiography/) and (exp artificial intelligence/ or artificial intelligence.ti,ab,kw. or exp deep learning/ or deep learning.ti,ab,kw. or exp machine learning/ or machine learning.ti,ab,kw. or exp artificial neural network/ or artificial intelligence.ti,ab,kw.)) NOT (ct OR computed tomography OR computed tomographic angiography).ti

#### Google Scholar

"coronary angiography" AND ("artificial intelligence"|"deep learning") -"ct" -"tomography"

The search was limited to studies written in English, human studies, and articles published in a peer-reviewed journal. The titles and abstracts of full text available articles were assessed on eligibility by a single researcher.

### Study Selection

ASReview selects relevant records by active learning, which is an interactive tool to train an algorithm with less data. Feature selection was performed with a natural language processing method called frequency-inverse document frequency. A Naïve Bayes classifier was iteratively trained to label records as relevant and non-relevant.
